# Efficient Overproduction of Membrane Proteins in *Lactococcus lactis* Requires the Cell Envelope Stress Sensor/Regulator Couple CesSR

**DOI:** 10.1371/journal.pone.0021873

**Published:** 2011-07-19

**Authors:** Joao P. C. Pinto, Oscar P. Kuipers, Ravi K. R. Marreddy, Bert Poolman, Jan Kok

**Affiliations:** 1 Groningen Biomolecular Sciences and Biotechnology Institute, Department of Molecular Genetics, University of Groningen, Groningen, The Netherlands; 2 Groningen Biomolecular Sciences and Biotechnology Institute, Netherlands Proteomics Centre and Zernike Institute for Advanced Materials, Department of Biochemistry, University of Groningen, Groningen, The Netherlands; Baylor College of Medicine, United States of America

## Abstract

**Background:**

Membrane proteins comprise an important class of molecules whose study is largely frustrated by several intrinsic constraints, such as their hydrophobicity and added requirements for correct folding. Additionally, the complexity of the cellular mechanisms that are required to insert membrane proteins functionally in the membrane and to monitor their folding state makes it difficult to foresee the yields at which one can obtain them or to predict which would be the optimal production host for a given protein.

**Methods and Findings:**

We describe a rational design approach to improve the lactic acid bacterium *Lactococcus lactis* as a producer of membrane proteins. Our transcriptome data shows that the two-component system CesSR, which senses cell envelope stresses of different origins, is one of the major players when *L. lactis* is forced to overproduce the endogenous membrane protein BcaP, a branched-chain amino acid permease. Growth of the BcaP-producing *L. lactis* strain and its capability to produce membrane proteins are severely hampered when the CesSR system itself or particular members of the CesSR regulon are knocked out, notably the genes *ftsH*, *oxaA2, llmg_2163* and *rmaB*. Overexpressing *cesSR* reduced the growth defect, thus directly improving the production yield of BcaP. Applying this rationale to eukaryotic proteins, some of which are notoriously more difficult to produce, such as the medically-important presenilin complex, we were able to significantly diminish the growth defect seen in the wild-type strain and improve the production yield of the presenilin variant PS1Δ9-H6 more than 4-fold.

**Conclusions:**

The results shed light into a key, and perhaps central, membrane protein quality control mechanism in *L. lactis*. Modulating the expression of CesSR benefited the production yields of membrane proteins from different origins. These findings reinforce *L. lactis* as a legitimate alternative host for the production of membrane proteins.

## Introduction

Membrane proteins comprise up to 30% of the proteome of any organism [1] and, in humans, are the direct targets of 60% of all pharmaceuticals [2]. Although the biological and medical relevance of membrane proteins is quite clear, they have been largely neglected due to a number of technical constraints, such as the production and purification of appropriate quantities of these proteins in their native form. This notion is driving many initiatives and international consortia [3] and has led to the development of novel ways of producing membrane proteins, such as cell-free expression systems [4]. However, the most reliable method to produce membrane proteins still consists of using cells to produce and insert them in the membrane in their native form. The bacterium *Escherichia coli* is the standard prokaryotic protein production host but, since membrane proteins encompass molecules that greatly differ with respect to structure, sugar decoration, lipid requirements and folding-factors needed, a broad set of hosts may have to be screened to find one best suited for production of a certain protein [5].

In addition to selecting specific hosts, attempts have been made to understand the intricacies of protein overproduction in a given host in order to improve it. Recently, based on a characterization of the *E. coli* BL21(DE3)-derived C41(DE3) and C43(DE3) strains, which are known for their increased ability to produce membrane proteins [6], Wagner *et al.* [7] designed *E. coli* Lemo21(DE3), a strain with a wider applicability due to the tunable activity of the T7 RNA polymerase driving the production of recombinant proteins. The elucidation of the response of *E. coli* to the production of membrane proteins has been used to design and re-engineer strains with improved membrane protein production capacity [8]–[10].

The fact that the spectrum of membrane proteins that can be produced by *Lactococcus lactis* is broadening justifies the consideration of this bacterium as a host for the overproduction of membrane proteins [11], [12]. Moreover, *L. lactis* is likely to provide a good membrane environment for proteins from the closely related pathogenic streptococci and enterococci. It is amenable to genetic manipulation and the paradigm for the broad clade of Lactic Acid Bacteria. Work with *L. lactis* profits of well-developed molecular biology protocols. High- and low-copy number stable plasmids as well as strong and tightly regulated promoter systems are available, allowing expression of toxic gene products [13]. Its relatively low proteolytic activity, single cytoplasmic membrane and an apparent failure to form inclusion bodies could make the production of membrane proteins more robust, easier to target and to purify than is the case for a number of other hosts, such as *E. coli*. The genomes of several *L. lactis* strains have been sequenced to completion: *L. lactis* subsp. *lactis* Il1403 [14], *L. lactis* subsp. *cremoris* SK11 [15], *L. lactis* subsp. *cremoris* MG1363 [16], *L. lactis* subsp. *cremoris* NZ9000 [17] and the plant-associated *L. lactis* subsp. *lactis* KF157 [18], enabling genome-wide studies such as transcriptome and proteome analyses. The small genome of the organism accounts for little redundancy, which facilitates complementation studies. Growth of *L. lactis* to high cell densities is rapid and does not require aeration, a feature that can save significant energy costs in industrial fermentations. *L. lactis* does not produce toxic/inflammatory compounds and, being Generally Regarded As Safe (GRAS) and widely used in food industry, should make it easier to market products of *e.g.*, therapeutic interest made with *L. lactis*.

Despite these advantages, *L. lactis* is not yet an ideal host for (membrane) protein overproduction. To further improve *L. lactis* in this respect and to generate enough protein to proceed with *e.g.*, structural studies, attempts should be made to rationally engineer improved protein production strains. Here, we present transcriptome data that reveal the response of *L. lactis* to the production of the endogenous cytoplasmic membrane protein BcaP, a branched-chain amino acid permease [19]. The involvement of the CesSR two-component system (TCS) and an analysis of its regulon suggest that this TCS monitors the integrity of the cell envelope and activates a response that facilitates the production of membrane proteins. The universality of this response is demonstrated in the accompanying paper, in which the transcriptome and proteome were analyzed in the context of stresses elicited by a range of membrane proteins [20]. This information was successfully used to rationally design *L. lactis* strains for improved functional production of (recombinant) membrane proteins of different types and origin, including eukaryotic ones.

## Results

### Functional overproduction of BcaP, BcaP-H6 and BcaP-GFP-H6 in *Lactococcus lactis*

**Figure 1:**
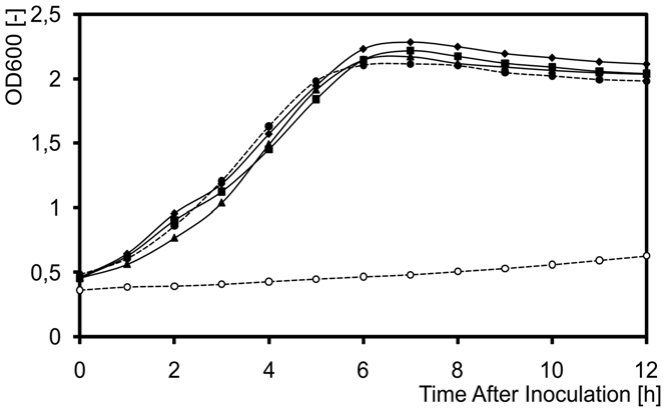
Functional production of BcaP and its derivatives in *L. lactis* MG1363Δ*bcaP*Δ*brnQ*. Strains were grown at 30°C in SA medium with all 20 amino acids. BcaP (full squares), BcaP-H6 (full diamonds) or BcaP-GFP-H6 (full triangles) were induced in either strain with 5 ng/m of nisin A at an OD_600_ = 0.4. The uncomplemented *L. lactis* MG1363Δ*bcaP*Δ*brnQ* is represented by a dotted line with empty circles and the uncomplemented wild-type *L. lactis* MG1363 by a dotted line with full circles.

*L. lactis* BcaP (Llmg_0118) is an approximately 50-kDa integral membrane protein. The protein, previously branded as CtrA, was recently renamed due to a better characterization of its function: it is involved in the internalization of branched-chain amino acids (BCA) in a process driven by the proton motive force [19]. BcaP is predicted to contain 12 transmembrane domains with a cytoplasmic C-terminus [21], allowing the fluorescence read-out of BcaP-GFP-H6 to be used as an indicator of correct folding of the chimeric protein [22], [23]. The functionality of the BcaP derivatives used in this study, carrying either a C-terminal hexa-histidine (H6) or a coupled GFP-H6 tag, was confirmed by their ability to restore the growth of *L. lactis* MG1363Δ*bcaP* (Fig. S1) and MG1363Δ*bcaP*Δ*brnQ* (Figure 1) in chemically defined SA media [24] with free amino acids as the only nitrogen source. In particular, *L. lactis* MG1363Δ*bcaP*Δ*brnQ* showed a severe growth defect in this medium as it cannot take up the essential BCAs due to the lack of the two dedicated transport systems, BcaP and BrnQ [19]. Complementation, *in trans*, with a copy of either chimeric version of BcaP restored the growth to wild type rates (Figure 1 and Fig. S1). All three BcaP variants were under the control of the nisin A-inducible *P_nisA_* promoter [13] and induction with nisin A in early exponential phase resulted in a complete overlap of the growth curves of the wild type and the complemented mutant strains.

**Figure 2:**
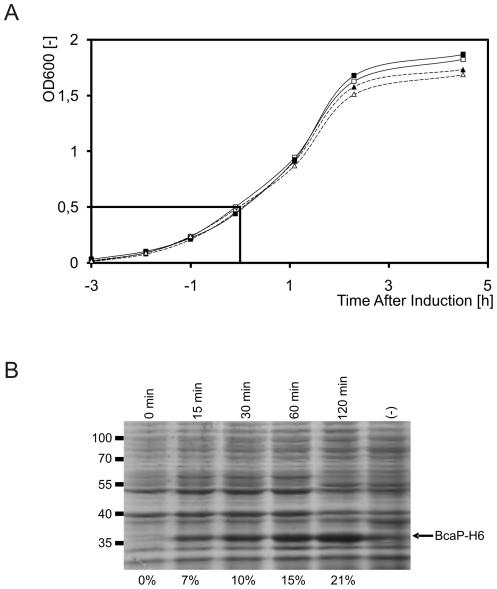
Production of BcaP-H6 in *L. lactis* NZ9000. A. Induction of cultures grown at 30°C in GM17 to an OD_600_ = 0.5 with 5 ng/m of nisin A, of *L. lactis* NZ9000 (pNZ*bcaP*-H6) (dotted lines and triangles) and the empty vector control *L. lactis* NZ9000 (pNZ8048) (dotted lines and squares). Open and closed symbols: independent biological replicates. B. Accumulation of BcaP-H6 in the membrane fraction, as observed in a Coomassie stained SDS-10% PAA gel. (-) denotes the empty vector control culture 2 hours after induction. The band indicated by the arrow was confirmed by imunoblotting to be BcaP-H6 (data not shown). Percentages indicate the relative concentration of BcaP-H6 in the membrane fraction at the indicated time points after induction. The data in panel B were obtained from one of the replicates in panel A.

When induced in a standard way, *i.e.* during the mid-exponential phase of growth (OD_600_ = 0.5), production of BcaP-H6 was easily achieved in *L. lactis* NZ9000 and only a minor effect on bacterial growth was observed (Figure 2A). Production of BcaP-H6 was quite significant already 15 min after induction and continued to rise steadily throughout. Two hours after induction, the overproduced BcaP-H6 accounted for 21% of the membrane protein fraction (Figure 2B). The overproduced BcaP-H6 was not clearly discernible using whole-cell-fractions on an SDS-PAA gel, suggesting that, at most, only a residual amount of the protein accumulated in the cytoplasm (data not shown).

### Genome-wide transcriptomic response of *L. lactis* to the overproduction of BcaP-H6

To understand the response of *L. lactis* to overproduction of BcaP-H6, we examined the transcriptome of *L. lactis* (pNZ8048::*bcaP-*H6) one hour after induction of the cells with nisin A, using full-genome DNA microarrays [25]. *L. lactis* (pNZ8048) was treated similarly to serve as the empty vector control (Figure 2A). Induction for one hour was used as it allowed for considerable amounts of BcaP-H6 to accumulate in the membrane (Figure 2B). Since growth was not significantly affected, the data is thought to mainly represent the direct response of *L. lactis* to the production and insertion of BcaP-H6 in the cytoplasmic membrane.

Table 1 shows that the CesSR regulon [26] constitutes a clear subset of genes in the group of genes that are significantly up-regulated in the BcaP-H6 overproducing strain. This further validates that CesSR may act as a sensor of the integrity of the cell envelope and, perhaps specifically, the cytoplasmic membrane [26]. Indeed, the CesSR-regulon genes encompass many strategies to react to various sources of cell envelope stress and to minimize its consequences. Notably, CesR induces expression of genes of which the products might counteract protein misfolding and assist in membrane protein biogenesis, such as *ftsH* (membrane-bound protease), *oxaA2* (pre-protein translocation component; YidC homologue) and *ppiB* (peptidyl-prolyl *cis-trans* isomerase).

**Table 1:** Effect of producing BcaP-H6 in *L. lactis* NZ9000 on the transcription of genes putatively belonging to the CesSR regulon.

Locus tag/gene	Description	ExpressionRatio	Bayesianp-value
*llmg_0021/ftsH*	Putative cell division protein	1.73	8.4*10^−5^
*llmg_0164/tgt*	Queuine tRNA-ribosyltransferase	1.68	1.6*10^−3^
*llmg_0165*	Predicted integral membrane protein	1.96	3.7*10^−4^
*llmg_0169*	Predicted integral membrane protein	4.23	1.5*10^−5^
*llmg_0540/oxaA2*	Preprotein translocase subunit	1.76	1.0*10^−4^
*llmg_0643/pacL*	Cation-transporting ATPase, E1–E2 family	2.25	3.0*10^−3^
*llmg_1103*	Putative uncharacterized protein	2.14	4.6*10^−5^
*llmg_1102*	Putative uncharacterized protein	1.74	3.4*10^−3^
*llmg_1101*	Putative secreted protein	2.46	1.9*10^−6^
*llmg_1115*	XpaC-like protein; Involved in cell morphology	10.3	2.1*10^−2^
*llmg_1116/telA1*	Toxic anion resistance protein	3.44	1.4*10^−4^
*llmg_1117/nagA*	N-acetylglucosamine-6-phosphate deacetylase	1.87	1.2*10^−3^
*llmg_1155/spxB*	Spx-like protein; Regulation of lysozyme resistance	5.60	5.3*10^−7^
*llmg_1156/yneG*	Putative transcriptional repressor	5.79	6.2*10^−5^
*llmg_1650*	Putative membrane protein	3.08	9.4*10^−7^
*llmg_1649/cesS*	Two-component system sensor (histidine kinase)	2.35	5.0*10^−5^
*llmg_1648/cesR*	Two-component system regulator	2.75	8.5*10^−7^
*llmg_1647*	Putative hydrolase from the Cof-subfamily	2.04	2.1*10^−5^
*llmg_1646/ppiB*	Peptidyl-prolyl *cis-trans* isomerase; Protein folding	2.32	4.0*10^−6^
*llmg_1645*	General stress protein GSP13	3.30	1.7*10^−6^
*llmg_1644*	Putative membrane protein	3.08	4.7*10^−5^
*llmg_1643/rodA*	Rod shape-determining protein	1.84	8.4*10^−5^
*llmg_1860/rmaB*	Transcriptional regulator from the MarR family	3.14	1.7*10^−6^
*llmg_1859*	Putative flavodoxin	2.58	9.1*10^−6^
*llmg_1857*	Putative esterase	1.94	2.0*10^−4^
*llmg_1856/lmrA*	Multidrug resistance ABC transporter ATP-binding and permease protein	1.83	3.7*10^−5^
*llmg_1918*	Putative membrane protein	2.55	2.9*10^−4^
*llmg_2164*	Putative uncharacterized protein	12.87	3.9*10^−10^
*llmg_2163*	Putative stress-responsive transcriptional regulator	11.88	2.5*10^−9^
*llmg_2420*	Putative uncharacterized protein	−1.29	9.7*10^−3^
*llmg_2477*	Lysine-specific permease	2.91	6.3*10^−6^

A full-genome DNA microarray analysis was performed, comparing *L. lactis* NZ9000 (pNZ*bcaP*-H6) and *L. lactis* NZ9000 (pNZ8048; empty vector control) (See Experimental Procedures). Cells were grown in GM17 and induced for 1 h with 5 ng/ml of nisin A once the OD_600_ was 0.5. The first putative members of operons are underlined. The minus sign on expression ratios indicates down-regulation in the strain producing BcaP-H6.

Other significantly differentially expressed genes (Table 2 and 3) can be taken together as the stringent stress response orchestrated via the (p)ppGpp allarmone pathway and is discussed in the accompanying paper [20], which also underpins the transcriptomics data presented here with proteomics results.

**Table 2:** Up-regulated expressed genes in *L. lactis* NZ9000 overproducing BcaP-H6 (for members of the CesSR regulon see Table 1).

Locus tag/gene	Description	Expression Ratio	Bayesian p-value
*llmg_0080/osmC*	Osmotically inducible protein C	2.43	3.9*10^−6^
*llmg_0099/rpmF*	50S ribosomal protein L32	1.86	1.3*10^−4^
*llmg_0098/rpmG*	50S ribosomal protein L33 1	2.76	1.2*10^−5^
*llmg_0100/cadA*	Cation-transporting ATPase	2.98	9.4*10^−6^
*llmg_0118/bcaP*	Branched-chain amino acid permease	95.73	1.7*10^−7^
*llmg_0124/secA*	Protein translocase subunit	1.39	2.5*10^−3^
*llmg_0138/argG*	Argininosuccinate synthase	2.17	1.3*10^−4^
*llmg_0139/argH*	Argininosuccinate lyase	6.29	5.8*10^−8^
*llmg_0180/cspE*	Cold shock-like protein; Transcription regulator	3.28	5.3*10^−6^
*llmg_0386/lysQ*	Amino-acid permease	2.40	3.8*10^−6^
*llmg_0410/groES*	Heat shock protein; 10 kDa chaperonin	2.75	1.2*10^−6^
*llmg_0411/groEL*	Heat shock protein; 60 kDa chaperonin	2.25	3.6*10^−6^
*llmg_0429/sodA*	Superoxide dismutase	2.47	8.6*10^−6^
*llmg_0527*	Putative uncharacterized protein	1.95	1.8*10^−5^
*llmg_0528/clpE*	ATP-dependent Clp protease ATP-binding subunit	2.21	1.1*10^−5^
*llmg_0535/gltS*	Arginine-binding periplasmic protein 1	2.37	8.2*10^−5^
*llmg_0536/argE*	Acetylornithine deacetylase	2.43	2.1*10^−5^
*llmg_0608/rpoE*	DNA-directed RNA polymerase subunit	2.05	9.4*10^−6^
*llmg_0638/clpP*	ATP-dependent Clp protease proteolytic subunit	2.31	2.6*10^−6^
*llmg_0868/tkt*	Transketolase	3.64	3.2*10^−7^
*llmg_0874/dapA*	Dihydrodipicolinate synthase	3.57	1.1*10^−6^
*llmg_0878/ffh*	Signal recognition particle protein	2.93	5.8*10^−7^
*llmg_0887*	Cation transport protein	2.30	6.9*10^−6^
*llmg_0986/clpB*	ATP-binding ClpB chaperone	2.78	2.5*10^−6^
*llmg_1204/hdiR*	HTH-type transcriptional regulator	2.20	9.5*10^−6^
*llmg_1208/rplL*	50S ribosomal protein L7/L12	3.27	9.8*10^−5^
*llmg_1275/aldB*	Alpha-acetolactate decarboxylase	1.87	2.4*10^−4^
*llmg_1274/aldR*	Putative regulator	3.15	4.2*10^−6^
*llmg_1555/whiA*	Putative transcription regulator	4.66	5.6*10^−7^
*llmg_1576/hrcA*	Heat-inducible transcription repressor	4.26	3.6*10^−7^
*llmg_1575/grpE*	Stress response protein; HSP-70 cofactor	4.30	1.7*10^−7^
*llmg_1574/dnaK*	Heat shock protein Hsp70; Chaperone	4.34	1.4*10^−7^
*llmg_1588/trxB1*	Thioredoxin reductase	1.71	1.3*10^−4^
*llmg_1587/secG*	Protein-export membrane protein	1.52	9.6*10^−3^
*llmg_1586/vacB1*	Putative exoribonuclease R	2.23	9.4*10^−5^
*llmg_1681/mraW*	S-adenosyl-methyltransferase	1.38	2.8*10^−1^
*llmg_1680/ftsL*	Cell division protein	1.58	3.0*10^−4^
*llmg_1679/pbpX*	Penicillin-binding protein	2.07	1.6*10^−4^
*llmg_1678/mraY*	Phospho-N-acetylmuramoyl-pentapeptide-transferase	2.61	5.0*10^−5^
*llmg_1744/ftsY*	Signal recognition particle-docking protein	2.19	9.4*10^−4^
*llmg_1756/argD*	Acetylornithine aminotransferase	3.89	2.1*10^−4^
*llmg_1755/argB*	Acetylglutamate kinase	4.45	3.7*10^−5^
*llmg_1754/argF*	Amino acid binding; Ornithine carbamoyltransferase	4.73	7.7*10^−5^
*llmg_1831/menB*	Naphthoate synthase	2.87	8.0*10^−7^
*llmg_2029/rplT*	50S ribosomal protein L20	2.24	4.3*10^−5^
*llmg_2172*	Putative nitroreductase	6.66	5.2*10^−8^
*llmg_2391*	Putative membrane protein; Acyltransferase	1.75	5.5*10^−5^
*llmg_2390/rpmGC*	50S ribosomal protein L33 3	1.84	3.0*10^−5^
*llmg_2389/secE*	Preprotein translocase SecE subunit	2.95	1.4*10^−3^

A full-genome DNA microarray analysis was performed, comparing *L. lactis* NZ9000 (pNZ*bcaP*-H6) and *L. lactis* NZ9000 (pNZ8048; empty vector control) (See Experimental Procedures). Cells were grown in GM17 and induced for 1 h with 5 ng/ml of nisin A once the OD_600_ was 0.5. The first putative members of operons are underlined.

**Table 3:** Down-regulated expressed genes in *L. lactis* NZ9000 overproducing BcaP-H6.

Locus tag/gene	Description	Expression Ratio	Bayesian p-value
*llmg_0336/plpB*	D-methionine-binding lipoprotein	−6.12	1.5*10^−8^
*llmg_0338/plpC*	D-methionine-binding lipoprotein	−9.16	1.8*10^−4^
*llmg_0339/dar*	Acetoin(diacetyl)reductase	−4.22	2.0*10^−7^
*llmg_0340/plpD*	D-methionine-binding lipoprotein	−2.78	1.7*10^−6^
*llmg_0341/metN*	Methionine import ATP-binding protein	−2.30	4.0*10^−6^
*llmg_0342*	Amino acid ABC transporter permease	−2.12	7.7*10^−6^
*llmg_0343*	Putative membrane protein	−2.72	6.8*10^−7^
*llmg_0344/cbiO*	Putative cobalt ABC transporter ATP-binding protein	−1.77	2.2*10^−4^
*llmg_501*	ABC transporter ATP binding protein	−5.52	5.1*10^−8^
*llmg_502*	ABC transporter permease protein	−4.65	8.3*10^−8^
*llmg_503*	Transcriptional regulator, LytR family	−3.25	2.7*10^−6^
*llmg_504*	Putative membrane protein	−2.57	1.1*10^−4^
*llmg_0588/kupB*	Potassium transport system protein	−2.09	6.1*10^−6^
*llmg_0650/brnQ*	Branched-chain amino acid transport system II	−3.26	2.0*10^−6^
*llmg_0697/oppD*	Oligopeptide transport ATP-binding protein	−5.50	2.2*10^−7^
*llmg_0698/oppF*	Oligopeptide transport ATP-binding protein	−5.81	2.4*10^−8^
*llmg_0699/oppB*	Peptide transport system permease protein	−5.50	3.1*10^−8^
*llmg_0700/oppC*	Oligopeptide transport system permease	−4.15	1.2*10^−7^
*llmg_0701/oppA*	Oligopeptide-binding protein	−3.23	6.1*10^−6^
*llmg_0702/pepO*	Endopeptidase O	−1.94	7.0*10^−5^
*llmg_0973/purC*	Phosphoribosylaminoimidazole-succinocarboxamide	−6.11	9.7*10^−6^
*llmg_0974/purS*	Phosphoribosylformylglycinamidine synthetase	−10.04	3.0*10^−6^
*llmg_0976/purL*	Phosphoribosylformylglycinamidine synthase 2	−5.63	7.1*10^−7^
*llmg_0994/purH*	Bifunctional purine biosynthesis protein	−5.61	3.8*10^−7^
*llmg_0997/purD*	Phosphoribosylamine-glycine ligase	−2.54	1.4*10^−4^
*llmg_0999/purE*	Phosphoribosylaminoimidazole carboxylase catalytic subunit	−2.19	3.0*10^−3^
*llmg_1000/purK*	Phosphoribosylaminoimidazole carboxylase ATPase subunit	−2.89	4.3*10^−4^
*llmg_1059/rsmC*	Ribosomal RNA small subunit methyltransferase C	−2.48	3.4*10^−6^
*llmg_1509/pyrE*	Orotate phosphoribosyltransferase	−2.40	3.7*10^−6^
*llmg_1508/pyrC*	Dihydroorotase	−1.43	3.8*10^−3^
*llmg_1532/ribD*	Riboflavin biosynthesis protein	−5.38	4.8*10^−8^
*llmg_1531/ribB*	Riboflavin biosynthesis protein	−7.28	7.2*10^−9^
*llmg_1530/ribA*	Riboflavin biosynthesis protein	−6.58	7.7*10^−8^
*llmg_1529/ribH*	6,7-dimethyl-8-ribityllumazine synthase	−2.88	6.9*10^−4^
*llmg_1541/nrdH*	Glutaredoxin-like protein nrdH	−2.92	6.0*10^−7^
*llmg_1542/nrdI*	Ribonucleotide reductase	−2.29	1.2*10^−5^
*llmg_1543/nrdE*	Ribonucleoside-diphosphate reductase	−2.95	9.7*10^−6^
*llmg_1544/nrdF*	Ribonucleoside-diphosphate reductase beta chain	−1.98	2.5*10^−5^
*llmg_1702/gshR*	Glutathione reductase	−1.90	7.1*10^−5^
*llmg_1700/choQ*	Choline ABC transporter ATP binding protein	−2.02	8.3*10^−6^
*llmg_1699/choS*	Choline ABC transporter permease and substrate binding protein	−1.64	2.7*10^−4^
*llmg_1776/metC*	ABC transporter permease protein	−4.89	8.7*10^−7^
*llmg_1775/cysK*	Cysteine synthase	−3.96	2.1*10^−6^
*llmg_1865/dtpT*	Di-/tripeptide transporter	−4.67	3.5*10^−7^
*llmg_1913/pbuO*	Xanthine/uracil/vitamin C permease	−8.45	4.5*10^−9^
*llmg_1912*	Putative uncharacterized protein	−3.41	8.9*10^−7^
*llmg_2026/oppB2*	Peptide transport system permease	−3.41	7.2*10^−6^
*llmg_2025/oppC2*	Oligopeptide transport system permease	−1.87	2.9*10^−1^
*llmg_2024/oppA2*	Oligopeptide-binding protein	−2.26	1.3*10^−4^
*llmg_2160/metK*	S-adenosylmethionine synthetase	−2.58	5.1*10^−6^
*llmg_2313/arcA*	Arginine deiminase	−1.83	1.1*10^−4^
*llmg_2312/arcB*	Ornithine carbamoyltransferase	−2.18	3.7*10^−6^
*llmg_2311/arcD1*	Arginine/ornithine antiporter	−2.57	6.4*10^−6^
*llmg_2310/arcC1*	Carbamate kinase	−2.49	2.7*10^−4^
*llmg_2309/arcC2*	Carbamate kinase	−1.91	2.4*10^−4^
*llmg_2361/secY*	Preprotein translocase subunit	−2.58	1.3*10^−6^

A full-genome DNA microarray analysis was performed, comparing *L. lactis* NZ9000 (pNZ*bcaP*-H6) and *L. lactis* NZ9000 (pNZ8048; empty vector control) (See Experimental Procedures). Cells were grown in GM17 and induced for 1 h with 5 ng/ml of nisin A once the OD_600_ was 0.5. The first putative members of operons are underlined. The minus sign on expression ratios indicates down-regulation in the strain producing BcaP-H6.

### The CesSR response is proportional to and follows, in time, the level of production of BcaP-*GFP-H6*

**Figure 3:**
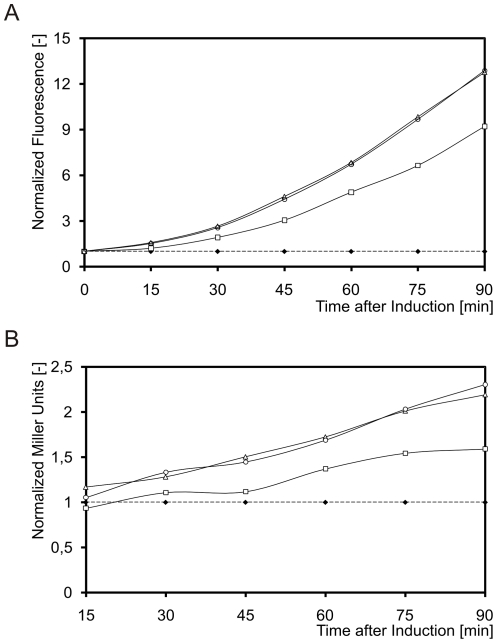
The CesSR response is proportional to, and follows in time, the level of production of BcaP-GFP-H6. The level of BcaP-GFP-H6 produced by *L. lactis* NZ9000 (pNZbcaP-GFP-H6; pAB0169) was modulated by inducing the strain at an OD_600_ = 0.5 with different amounts of the inducer nisin A: 0 ng/ml (control; dotted line and full diamonds), 0.25 ng/ml (empty squares), 2.5 ng/ml (empty triangles), 25 ng/ml (empty circles). A. Average BcaP-GFP-H6 fluorescence per cell. B. β–galactosidase activity specified by the P*llmg_0169*-*lacZ* fusion in plasmid pAB0169. In both A and B values were normalized to the uninduced culture in both figures.

We characterized the kinetics of activation of CesSR in relation to the production and insertion of BcaP-GFP-H6 in the cytoplasmic membrane. Determining whole-cell-fluorescence enables directly estimating the amount of correctly folded BcaP-GFP-H6 [22], [23]. Since fluorescence is dependent on the proper insertion of the entire chimeric protein in the cytoplasmic membrane, its quantification measures the production burden in terms of the amount of recombinant membrane proteins allocated in the membrane. *L. lactis* NZ9000 (pNZ*bcaP*-GFP-H6; pAB0169) was used in these experiments. Plasmid pAB0169 carries a fusion of *lacZ* from *E. coli* to the promoter of *llmg_0169*, a gene that is activated by CesSR and serves as an indicator of CesSR activity [26]. Expression of the CesSR regulon was immediate; its level of up-regulation was highly correlated to the level of BcaP-GFP-H6 present in the cytoplasmic membrane at any given moment (Figure 3). Similar data was obtained using a *lacZ* fusion to the promoter of *llmg_2164*, another CesSR regulon member (data not shown).

We also observed an increased growth defect, albeit relatively small, when more of the inducer nisin A was used (data not shown). Growth rate differences did not further activate CesSR, since using either 2.5 ng/ml or 25 ng/ml of nisin A yielded equivalent β-galactosidase activity profiles. Also, no increased fluorescence was detectable in those cells. This supports the conclusion that the level of activation of CesSR was exclusively a consequence of the amount of BcaP-GFP-H6 in the membrane and not, unlike many other stress responses, linked to a hampered growth rate.

### The CesSR regulon comprises genes that are essential for membrane protein overproduction

**Figure 4:**
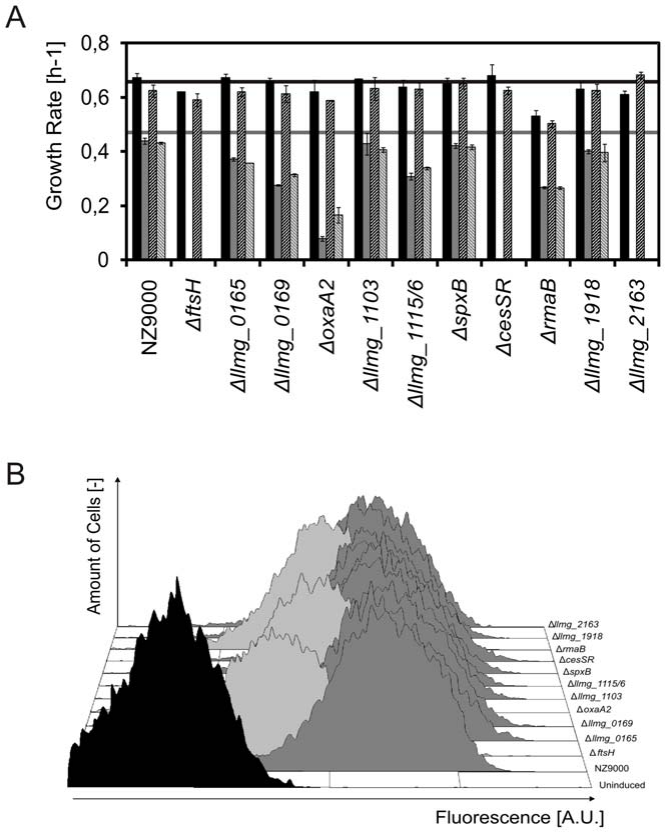
Phenotypic characterization of *L. lactis* strains carrying knock-out deletions in genes from the CesSR regulon. A. Growth rates in GM17 of the indicated deletion strains carrying pNZ*bcaP*-H6 (producing BcaP-H6; full bars) or carrying pNZ*bcaP*-GFP-H6 (producing BcaP-GFP-H6; striped bars) when uninduced (black or black and white stripes) or induced with 5 ng/ml nisin A (gray or gray and white stripes) at OD_600_ = 0.5. The horizontal lines represent growth of the empty vector control strain *L. lactis* NZ9000 (pNZ8048) (uninduced – dark line; induced – gray line). B. Distribution, as measured by flow-cytometry, of BcaP-GFP-H6 content per cell 1 hour after induction of the protein from pNZ*bcaP*-GFP-H6 carried by the indicated knock-out mutants.

To examine whether genes induced after overproduction of membrane protein via CesR could constitute a mechanism(s) to cope with the added burden, in-frame and non-polar knock-out mutants of *L. lactis* NZ9000 were made for 11 genes in the regulon (Table 4). As all mutants could be made, none of these genes were essential under the conditions used here. The only significant phenotypic difference observed was an approximately 20% decrease of the growth rate of *L. lactis* Δ*rmaB* in glucose-supplemented M17 medium (Figure 4A).

**Table 4:** Strains used in this study.

Strain	Description	Reference or Source
***L. lactis***		
MG1363	*L. lactis* subsp. *cremoris*, plasmid-free derivative of NCDO712	[50]
MG1363ΔbcaP	MG1363 derivative with chromosomal deletion of *bcaP*	[19]
MG1363ΔbcaPΔbrnQ	MG1363 derivative with chromosomal deletion of bcaP and *brnQ*	[19]
NZ9000	MG1363Δ*pepN::nisRK*	[51]
*ΔftsH*	NZ9000 derivative with chromosomal deletion of *ftsH*	This work
*Δllmg_0165*	NZ9000 derivative with chromosomal deletion of *llmg_0165*	This work
*Δllmg_0169*	NZ9000 derivative with chromosomal deletion of *llmg_0169*	This work
*ΔoxaA2*	NZ9000 derivative with chromosomal deletion of *oxaA2*	This work
*Δllmg_1103*	NZ9000 derivative with chromosomal deletion of *llmg_1103*	This work
*Δllmg_1115/6*	NZ9000 derivative with chromosomal deletion of *llmg_1115* and *llmg_1116*	This work
*ΔspxB*	NZ9000 derivative with chromosomal deletion of *spxB*	This work
*ΔcesSR*	NZ9000 derivative with chromosomal deletion of *cesS* and *cesR*	This work
*ΔrmaB*	NZ9000 derivative with chromosomal deletion of *rmaB*	This work
*Δllmg_1918*	NZ9000 derivative with chromosomal deletion of *llmg_1918*	This work
*Δllmg_2163*	NZ9000 derivative with chromosomal deletion of *llmg_2163*	This work
***E. coli***		
DH5α	Cloning host	Bethesda Research Laboratories

The plasmid pNZ*bcaP*-GFP-H6 was introduced in all mutants and production of BcaP-GFP-H6 was induced with nisin A. The growth rates of strains Δ*ftsH*, Δ*oxaA2*, Δ*cesSR* and Δ*llmg_2163* were severely affected upon the induction, while the strains Δ*llmg_0165*, Δ*llmg_0169*, Δ*llmg_1115/6* and Δ*rmaB* were partially impaired (Figure 4A). The remainder of the strains (Δ*llmg_1103*, Δ*spxB* and Δ*llmg_1918*) grew like the control strain NZ9000 (pNZ*bcaP*-GFP-H6). Similar results were obtained when BcaP-H6 was overexpressed in the mutant strains, demonstrating that the GFP moiety of the fusion protein was not responsible for these observations (Figure 4A). Adding nisin A to plasmid-free knock-out strains did not lead to changes in growth (data not shown), thus excluding the influence of the inducer, *per se*.

Production of BcaP-GFP-H6 in the knock-out strains was measured as whole-cell fluorescence using flow-cytometry. While no significant differences were observed for most mutants, the Δ*ftsH*, Δ*oxaA2* and Δ*rmaB* strains were greatly affected in their capacity to produce BcaP-GFP-H6 (Figure 4B).

### Overexpression of cesSR improves the capability of *L. lactis* to produce membrane proteins

Given that some genes from the CesSR regulon are, apparently, critical in membrane protein production, we examined whether cell fitness could be improved by modulating expression of those genes, namely *ftsH*, *oxaA2*, *cesSR*, *rmaB* and *llmg­_2163*. The promoterless genes were cloned in pIL252 and pIL253 (low- and high-copy number vectors, respectively). Constitutive transcription of these genes in both plasmids was achieved by read-through from the plasmid replication genes [27]. The resulting plasmids were introduced in the corresponding *L. lactis* mutant strains, which already carried plasmid pNZ*bcaP*-GFP-H6.

**Figure 5:**
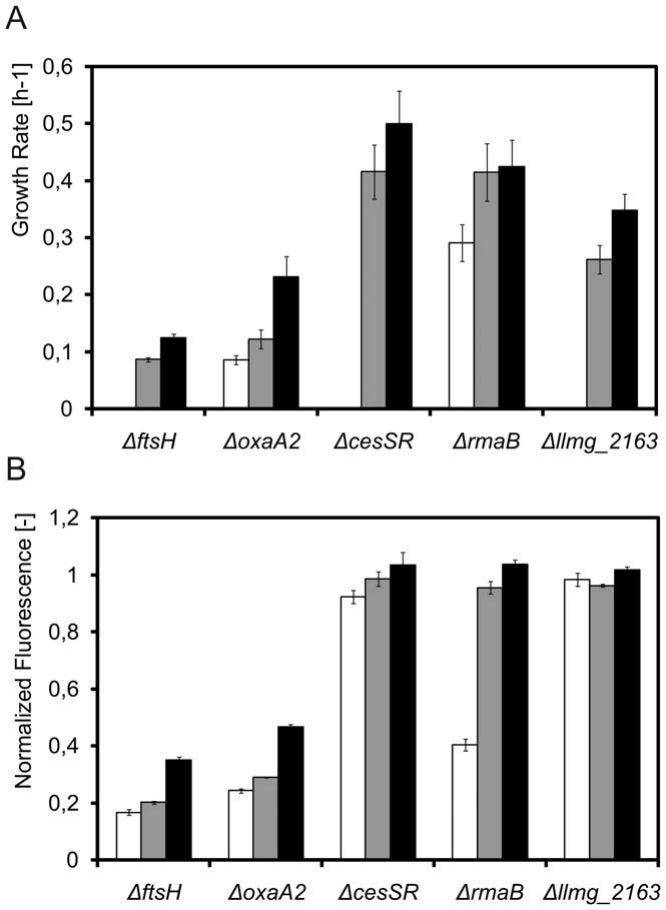
Complementation of *L. lactis* knock-out strains. Complementation of *L. lactis* knock-out strains by *in trans* expression of the corresponding genes from pIL252 (gray) or pIL253 (black) derivatives. Data for the uncomplemented knock-out mutants is shown in white. All strains contained plasmid pNZ*bcaP*-GFP-H6, for nisin A-inducible expression of BcaP-GFP-H6. The growth rate (A) and the ability of the knock-out strains to produce BcaP-GFP-H6 (B) after induction with 5 ng/ml at an OD_600_ = 0.5 was examined at 30°C in GM17. Fluorescence values in B were normalized to the wild type, *L. lactis* NZ9000 (pNZ*bcaP*-GFP-H6).

*In trans* complementation of the mutations with the matching pIL253 and pIL252 derivatives restored growth (Figure 5A) and the capability to produce BcaP-GFP-H6 (Figure 5B) to the wild type level for the complemented Δ*cesSR* and Δ*rmaB* strains, almost fully for Δ*llmg_2163*, but only partially for the Δ*ftsH* and Δ*oxaA2* mutants. In all cases the use of the low copy-number pIL252 derivatives corresponded to an intermediate phenotype, showing that the genes were being expressed at different levels as a result of the different plasmid copy numbers.

**Figure 6:**
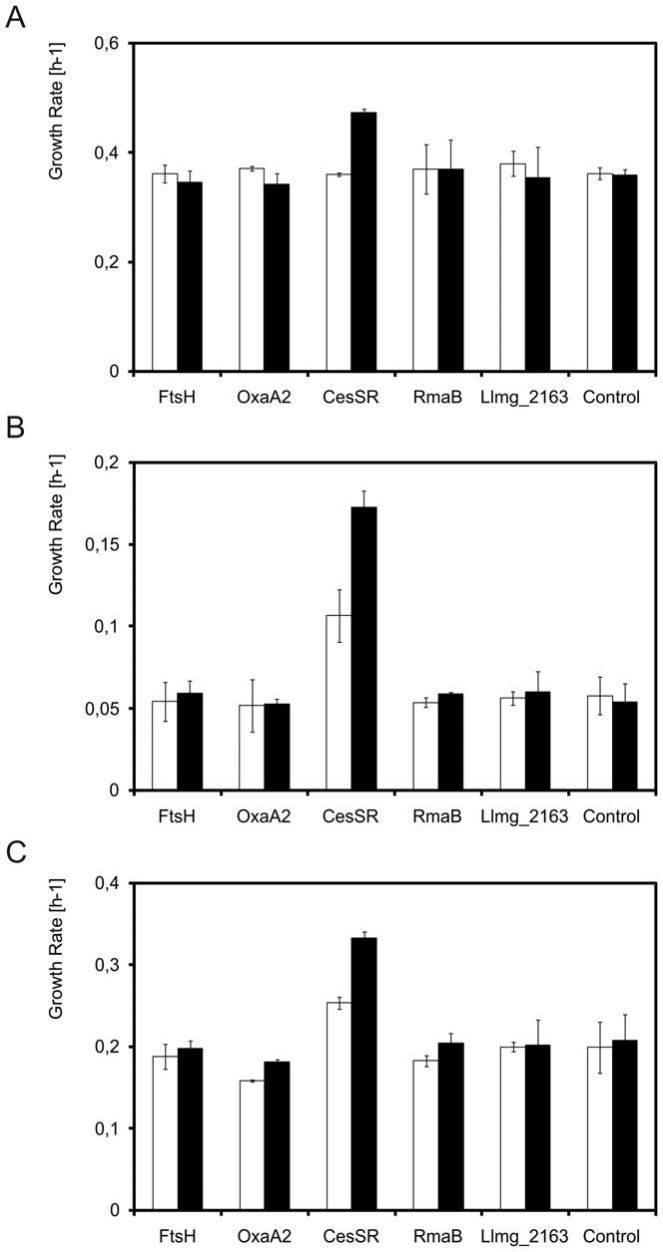
Production of eukaryotic membrane proteins in *L. lactis* NZ9000. Effect on growth at 30°C in GM17 with appropriate antibiotics of the simultaneous production of membrane protein (A. BcaP-H6; B. PS1Δ9-H6; C. StSUT1-H6) and members of the CesSR regulon, as indicated on the abscissa. The *L. lactis* NZ9000 strains carried 2 plasmids, one was a pNZ8048 derivative containing the nisin A-inducible gene of the membrane protein in question, the other was either a pIL252 (white bars) or a pIL253 (black bars) derivative from which the CesSR regulon gene was constitutively expressed (see Table 5). The membrane protein genes were induced with 5 ng/ml nisin A when the cultures had reached and OD_600_ = 0.5. Control in all three panels: *L. lactis* NZ9000 (pNZ8048) carrying either pIL252 or pIL253.

Subsequently, the various pIL252 and pIL253 derivatives were introduced in *L. lactis* NZ9000 (pNZ*bcaP*-GFP-H6). Using the standard induction concentration of 5 ng/ml of nisin A at an OD_600_ = 0.5 did not yield any differences in the production of BcaP-GFP-H6 between these strains, as measured by flow-cytometry (data not shown). Although differences in the average fluorescence per cell were not observable, *L. lactis* NZ9000 (pNZ*bcaP*-GFP-H6; pIL253::*cesSR*) showed a 30% improved growth rate (Figure 6A).

Several other proteins, especially from eukaryotic origin, are produced to a much lesser extent in *L. lactis* compared to what we document here for BcaP [11]. We chose two relatively poorly produced proteins – a variant of presenilin missing exon 9 (PS1Δ9), which is linked to an increased susceptibility to Alzheimer [28], and a sucrose transporter from *Solanum tuberosum* (StSUT1) [29]. Both proteins were hexa-histidine-tagged at their C-termini. The construction of both nisin A-inducible overexpression constructs is described in the accompanying paper [20]. The presence of pIL253::*cesSR* in *L. lactis* strains in which PS1Δ9-H6 or StSut1-H6 overexpression was induced with nisin A resulted in over 3-fold (PS1Δ9-H6; Figure 6B) or over 1.5-fold (StSUT1-H6; Figure 6C) increase in the growth rates of the induced strains. The presence of the lower copy pIL252::*cesSR* caused an intermediate growth improvement after induction, but still 2-fold for PS1Δ9-H6 (Figure 6B).

**Figure 7:**
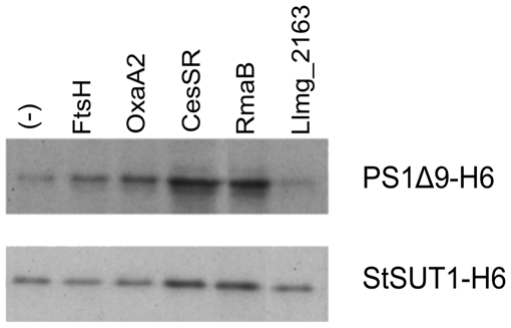
Production of PS1Δ9-H6 and StSUT1-H6 in *L. lactis* NZ9000. Top, *L. lactis* NZ9000 (pNZPS1Δ9) strains. Bottom, *L. lactis* NZ9000 (pNZStSUT1) strains. Each strain carried, in addition to the indicated plasmid, a pIL253 derivative from which the protein specified at the top of the figure was constitutively co-expressed. Cells were grown at 30°C in GM17 and induced for 1 h with 5 ng/ml nisin A at an OD_600_ = 0.5. The membrane protein fraction was obtained and separated on an SDS-(10%)PAA gel. The gel was immunoblotted and examined for the presence of PS1Δ9-H6 and StSUT1-H6 using anti-His antibodies.

Co-expression of *cesSR* also directly influenced production of PS1Δ9-H6 and StSUT1-H6 (Figure 7). Strains carrying pIL253::*cesSR* produced four times more PS1Δ9-H6 or two times more StSUT1-H6 than the control strain with the empty pIL253 vector. Despite not having any perceptible effect on the growth, pIL253::*rmaB* proved to be equally capable of aiding in the production of these eukaryotic proteins. Co-producing FtsH or OxaA2 also improved membrane protein production, although not to the same extent as CesSR or RmaB did, and only so for PS1Δ9-H6. We did not detect any significant improvement in PS1Δ9-H6 or StSUT1-H6 production with any of the pIL252 derivatives (data not shown).

## Discussion

Studies on membrane proteins are greatly constrained by a number of technical obstacles, of which production of sufficient quantities in their native form is arguably the major one. To allow designing strains for the dedicated purpose of producing membrane proteins, we characterized the stress that *L. lactis* cells endure during the production of high amounts of these proteins.

*L. lactis* can efficiently produce the endogenous branched-chain amino acid permease BcaP and its derived tagged variants, BcaP-H6 and BcaP-GFP-H6. All three proteins accumulate in the cytoplasmic membrane to up to 20% of all membrane proteins in only two hours after induction of their production.

Transcriptome analysis of the response evoked on *L. lactis,* while producing BcaP-H6, revealed that CesSR is highly activated. This finding gives further credit to the idea that this two-component system (TCS) responds to cell envelope stresses. Previously, CesSR was shown to be triggered when cells are treated with lipid II-interacting cationic polypeptides, while disruption of *cesR* led to an increased susceptibility to such membrane-active antimicrobials [26]. It has also been shown that *L. lactis* CesSR modulates resistance of peptidoglycan to hydrolysis via induction of *spxB* [30]. CesSR is homologous to *Bacillus subtilis* and *Streptococcus mutans* LiaRS [31], [32] and *Staphylococcus aureus* VraSR [33], [34], all of which are also involved in the response to cell envelope stresses. In these, and probably in many pathogenic bacteria, this response is linked to resistance to several antibiotics, such as β-lactams and vancomycin.

To the best of our knowledge, there has been no report of CesSR (or a homologous TCS) being activated during production of cytoplasmic proteins, a change in the culture pH, temperature, salt concentration or the redox state of the medium (Anne de Jong, personal communication), thus confirming the specificity of this response to cell envelope stresses.

In the apparent absence of stress response-dedicated sigma factors in *L. lactis*, some of their roles have most likely been taken over by TCSs. Eight putative TCSs have been identified in *L. lactis* MG1363 [16], six of which have been proven to be necessary for normal growth or to be involved in resistance to several kinds of aggressions [35]. Further support for the importance of CesSR in *L. lactis* comes from the relative size of its regulon. It contains more than 30 genes in *L. lactis* while only two promoters are known to be regulated by LiaRS in *B. subtilis* [36].

Our transcriptome data supports the previous description of the CesSR regulon with respect to its members as well as their relative induction levels [26], despite imposing a completely different stress on the cells. Our results give little support for *llmg­_2420*, encoding a putative glycosyl transferase, being regulated by CesRS. The genes *ftsH* (*llmg_0021*) and *tgt* (*llmg_0164*), both of which contain a putative CesR motif in their promoter region, were up-regulated in our study and are therefore likely members of the regulon. Also, our data indicates that the operon *llmg_1115-llmg_1116* might also include *llmg_1117* and that the operon *llmg_1650-llmg_1646* (comprising *cesSR*) might also contain *llmg_1645*, *llmg_1644* and *llmg_1643*.

It should be noted that the vast majority of genes regulated by CesSR are either putative membrane proteins or proteins directly acting on the membrane. Overall, it seems that this stress response provides *L. lactis* with a mechanism(s) to cope with cell envelope damage. Remarkably, some members of the regulon, such as *ftsH*, *oxaA2* and *ppiB*, code for proteins that assist in membrane protein biogenesis and quality control, a strategy that might explain the high production yields of BcaP-H6 in *L. lactis*.

Other differentially-expressed general stress response genes, such as *hrcA-grpE-dnaK*, *groES-groEL*, *clpE*, *clpP*, *clpB*, *sodA*, *cspE*, are indicative of an increased number of mis- or unfolded proteins in the cell. These could be BcaP-H6 molecules that are not able to insert correctly into the membrane or, due to the high turnover, get misfolded. Additionally, the chaperones and translocation machinery used for BcaP-H6 production and insertion in the membrane might become overloaded at high production rates, leading to an increase of misfolding of other proteins.

A burden on the translocation pathway is reflected in the up-regulation of *ffh*, *ftsY*, *secG*, *secA*, *secE* and the above mentioned *ftsH* and *oxaA2*. Apparently, the cell attempts to upgrade the capacity to insert proteins in the cytoplasmic membrane. The down-regulation of *secY* seems to be in obvious contradiction. Possibly, *L. lactis* tries to adapt by manipulating the relative influence of specific translocation pathways, such as YidC and/or SecYEG independently, co-translationally or not. Translocation did not appear to be a putative bottleneck when *L. lactis* was induced to produce CFTR [37], a eukaryotic protein for which only trace amounts could be obtained. The up-regulation of members of the translocation pathway only when substantial amounts of recombinant membrane protein are produced suggests that translocation may represent a bottleneck in these situations.

BrnQ, a branched-chain amino acid transporter, and other peptide transport systems such as OppB2C2A2, OppDFBCA-PepO, MetN and DtpT were highly down-regulated, possibly as a result of the almost 100-fold up-regulation of *bcaP*-H6 relative to the endogenous *bcaP* in the control strain. It is likely that the native *bcaP* gene is also differentially expressed in the overproducing strain, although this could not be assessed as the dedicated DNA microarray probe does not distinguish between *bcaP* and *bcaP*-H6. Many other metabolic processes, such as the arginine, purine and pyrimidine biosynthetic pathways, were affected when *L. lactis* overproduced BcaP-H6. Transcription, through *rpoE*, and translation, through the differential expression of many genes coding for ribosomal proteins, also seem to be affected in the overproducing strain. Some of these observations might have been caused by differences in the growth rate, however small, between the induced strain and its control.

The kinetics and intensity of the CesSR-mediated response to membrane protein overproduction was examined using *lacZ* fusions to the promoters of *llmg_0169* and *llmg_2164*, while the fluorescent signal from single cells producing BcaP-GFP-H6 was used as a measure for the amount of correctly folded protein. The activation of the CesSR response was fully related to the amount of correctly folded proteins in the cytoplasmic membrane, even when induction started to visibly affect cellular growth. This observation is in accordance with the fact that other stresses, even the burden of overproducing cytoplasmic proteins, do not trigger CesSR (see accompanying paper [20] and Anne de Jong, personal communication).

Of 11 clean deletion mutants for CesSR regulon genes that were considered to be most relevant to the response, both based on their (putative) function and their fold up-regulation in our transcriptome data, none were affected under during normal growth conditions. Most of them, however, displayed growth problems under BcaP-H6 or BcaP-GFP-H6 overproduction stress. In particular, the growth of strains lacking *ftsH*, *cesSR* or *llmg_2163* stopped completely under these conditions, a clear indication that CesSR activates a mechanism(s) that allows *L. lactis* to cope with the added burden. Moreover, *L. lactis* strains Δ*ftsH*, Δ*oxaA2* and Δ*rmaB* were greatly affected in their capacity to produce BcaP-GFP-H6. This observation indicates that all of these genes need to be expressed to some extent already at the moment of induction and in a CesSR-independent manner, since the Δ*cesSR* strain did not show the same phenomenon. OxaA2, homologous to YqjG from *B. subtilis*, proved to be essential only when cells are required to translocate unusually high amounts of protein. An *L. lactis* OxaA2 orthologue, Llmg_0143 (similar to *B. subtilis* SpoIIIJ) thus seems sufficient for the default translocation rate, provided that both proteins OxaA2 and Llmg_0143 are exchangeable, as has been shown to be the case in *B. subtilis* [38]. It remains to be examined whether an *L. lactis* Δ*llmg_0143* strain has a phenotype comparable to that of *L. lactis* Δ*oxaA2*. The role of FtsH in the quality control of membrane proteins explains the observed phenotype when *L. lactis* overproduces BcaP-GFP-H6. During normal growth of *L. lactis* Δ*ftsH*, FtsH is not essential as cells probably have redundant mechanisms to correct normal levels of disorganization and misfolding of proteins in the cytoplasmic membrane. Unlike previously theorized for *E. coli* [8], *L. lactis* Δ*ftsH* is handicapped when forced to produce high amounts of membrane proteins.

*L. lactis* RmaB is a putative regulator of the MarR family [16]. *L. lactis* Δ*rmaB* was the only mutant displaying a clear phenotype during normal growth conditions: it grew 20% slower than the wild type. Upon induction of BcaP-H6 overproduction, *L. lactis* Δ*rmaB* displayed only a relatively small further increase in its growth defect but proved to be as limited in its capacity to produce BcaP-H6 as *L. lactis* Δ*cesSR*. These observations and the putative implication of RmaB in non-specific multiple antibiotic resistance, being a regulator of the MarR family, suggest that this regulator might contribute to the general fitness of the cell envelope.

*L. lactis* strains Δ*cesSR* and Δ*llmg_2163* showed, *per se*, no perceptible handicap to overproduce BcaP-GFP-H6 and both genes were necessary only to sustain bacterial growth after induction. This shows that CesSR is required only when a stress response has to be triggered to cope with the added burden. This seems to be accomplished by activating and adjusting quality control mechanisms and translocation capacity. Llmg_2163, probably a transcriptional regulator since it contains a putative PspC DNA binding domain [39], might further enhance this cell envelope stress response.

By cloning *ftsH*, *oxaA2*, *cesSR*, *rmaB* and *llmg_2163* into high- and low-copy number vectors, their influence on membrane protein production could be assessed under six different levels of expression (two different plasmids and an empty vector control in two different backgrounds *i.e.,* the wild-type and deletion strains). As pIL252/pIL253 derivatives have a replicon that is different from that of pNZ8048 (see Table 5), the expression of the CesSR regulon genes could be examined simultaneously with the production of a membrane protein using a two-vector approach. Complementation of the mutant strains with their corresponding genes on pIL252 and pIL253 was obtained, but only partially for *ftsH* and *oxaA2*. Possibly, the native expression of these two genes is higher than what we were able to obtain with the plasmid constructs. In all cases, the pIL253 derivatives performed better at restoring the wild type phenotypes.

**Table 5:** Plasmids used in this study.

Plasmid	Description	Reference or Source
pNZ8048	Cm^r^; Expression vector with nisin A-inducible P_nisA_	[52]
pNG*bcaP*	pNZ8048 containing *bcaP* downstream of P_nisA_	[19]
pNG*bcaP*-H6	pNZ8048 containing the *bcaP*-H6 gene downstream of P_nisA_; for expression of BcaP with 6His-tag at the C-terminus	[19]
pNZcLIC	pNZ8048 derivative that enables cloning by the LIC-VBEx procedure	[53]
pNZ*bcaP*-GFP-H6	pNZcLIC derivative containing the *bcaP-GFP-H6* gene downstream of PnisA; for expression of BcaP with GFP-H6 at the C-terminus	[23]
pNZPS1Δ9	pNZcLIC derivative containing the *PS1Δ9-H6* gene downstream of PnisA; for expression of PS1 Δ9 with 6His-tag at the C-terminus	[20]
pNZStSUT1	pNZcLIC derivative containing the *StSut1-H6* gene downstream of PnisA; for expression of StSUT1 with His-tag at the C-terminus	[20]
pAB0169	Tet^r^; pPTL derivative carrying the promoter of *llmg_0169*	[26]
pAB2164	Tet^r^; pPTL derivative carrying the promoter of *llmg_2164*	[26]
pCS1966	Em^r^; *oroP*; 5-fluoroorotate selection/couterselection vector for chromosomal integration in L. lactis	[43]
pCS1966::*ftsH*	pCS1966 derivative for deletion of chromosomal *ftsH*	This Work
pCS1966::*llmg_0165*	pCS1966 derivative for deletion of chromosomal *llmg_0165*	This Work
pCS1966::*llmg_0169*	pCS1966 derivative for deletion of chromosomal *llmg_0169*	This Work
pCS1966::*llmg_0540*	pCS1966 derivative for deletion of chromosomal *oxaA2*	This Work
pCS1966::*oxaA2*	pCS1966 derivative for deletion of chromosomal *llmg_1103*	This Work
pCS1966::*llmg_1115*/6	pCS1966 derivative for deletion of chromosomal *llmg_1115/6*	This Work
pCS1966::*cesSR*	pCS1966 derivative for deletion of chromosomal *cesSR*	This Work
pCS1966::*rmaB*	pCS1966 derivative for deletion of chromosomal *rmaB*	This Work
pCS1966::*llmg_1918*	pCS1966 derivative for deletion of chromosomal *llmg_1918*	This Work
pCS1966::*llmg_2163*	pCS1966 derivative for deletion of chromosomal *llmg_2163*	This Work
pVE6007	Cm^r^; Replication-thermosensitive derivative of pWV01	[54]
pVES4196	Em^r^; pORI280 derivative for deletion of chromosomal *llmg_1155*	[30]
pIL252	Em^r^; low copy number derivative of pAMβ1	[27]
pIL252::*ftsH*	pIL252 derivative expressing *ftsH*	This Work
pIL252::*oxaA2*	pIL252 derivative expressing *oxaA2*	This Work
pIL252::*cesSR*	pIL252 derivative expressing *cesSR*	This Work
pIL252::*rmaB*	pIL252 derivative expressing *rmaB*	This Work
pIL252::*llmg_2163*	pIL252 derivative expressing *llmg_2163*	This Work
pIL253	Em^r^; high copy number derivative of pAMβ1	[27]
pIL253::*ftsH*	pIL253 derivative expressing *ftsH*	This Work
pIL253::*oxaA2*	pIL253 derivative expressing *oxaA2*	This Work
pIL253::*cesSR*	pIL253 derivative expressing *cesSR*	This Work
pIL253::*rmaB*	pIL253 derivative expressing *rmaB*	This Work
pIL253::*llmg_2163*	pIL253 derivative expressing *llmg_2163*	This Work

*oroP*: Fluoroorotate transporter gene.

Em^r^: Erythromycin resistance marker.

Cm^r^: Chloramphenicol resistance marker.

Tet^r^: Tetracycline resistance marker.

P*_nisA_*: native promoter of *nisA*.

*L. lactis* NZ9000 (pIL253::*cesSR*; pNZ*bcaP*-GFP-H6), overproducing BcaP-GFP-H6 in the presence of extra plasmid copies of *cesSR*, could be singled out for its improved growth rate. However, no significant improvement was detected for BcaP-GFP-H6 production per cell, probably as a consequence of the fact that the fusion protein, two hours after induction, is already produced at very high levels of over 20% of all proteins in the membrane fraction.

When *L. lactis* is forced to make eukaryotic proteins, *e.g.* PS1Δ9-H6 and StSUT1-H6, which are known to be very difficult to produce [20], pIL252::*cesSR* and pIL253::*cesSR* highly increased the growth rate of the producer strains, leading to a substantial increase in protein production. This result demonstrates the importance of the orchestrating role of CesSR, since over-expression of any other particular gene alone did not improve growth. Immunoblotting also showed that PS1Δ9-H6 and StSUT1-H6 were better produced during co-expression of *cesSR*. Although it did not improve growth, pIL253::*rmaB* was equally capable of improving the production of the two eukaryotic proteins. While the increased production capacity of the strain carrying pIL253::*cesSR* might be partially explained through an up-regulation of *rmaB*, the improved growth rate of that strain remains unexplained since none of the other vectors produced an equivalent result. It seems likely that cells overproducing CesSR benefit from the sum of all partial contributions of all, or a subset of, the members of the CesSR regulon.

## Materials and Methods

### Bacterial strains, plasmids and growth conditions

Strains and plasmids used in this study are listed in Tables 4 and 5, respectively. *L. lactis* was grown at 30°C in M17 medium (Difco laboratories, Detroit, MI) supplemented with 0.5% (wt/vol) glucose (GM17) as standing cultures or on GM17 agar plates containing 1.5% (wt/vol) agar. The chemically defined medium SA, in which free amino acids serve as a nitrogen source, was prepared and used as previously described [24]. When required, chloramphenincol (Cm), erythromycin (Em) or tetracycline (Tc) (all from Roche Molecular Biochemicals, Mannheim, Germany) were added to media at 5 µg ml^−1^. When two antibiotics were used, the concentration of each was reduced to 2.5 µg ml^−1^. All growth rates were calculated following OD_600_ readings with an Infinite F200 microtiter plate reader (Tecan, Grödig, Austria). Induction with nisin A (Sigma-Aldrich, St. Louis, MO) was done with 5 ng/ml, unless stated otherwise.

### DNA isolation and manipulation

Routine molecular cloning techniques were performed essentially as described by Sambrook and Russel [40]. Total chromosomal DNA from *L. lactis* was extracted according to Johansen and Kibenich [41]. Mini-preparations of plasmid DNA from *L. lactis* were done using the High Pure Plasmid isolation kit (Roche Molecular Biochemicals). Oligonucleotides were synthesized by Biolegio (Biolegio BV, Nijmegen, the Netherlands). PCR products were amplified using Phusion DNA polymerase (Finnzymes Oy, Keilaranta, Finland) according to manufacturer's instructions. PCR products were purified using the High Pure PCR product purification kit (Roche Molecular Biochemicals). Restriction enzymes and T4 DNA ligase were purchased from Fermentas (Ontario, Canada). Plasmid DNA was introduced into *L. lactis* by electrotransformation, using a Bio-Rad Gene Pulser (Bio-Rad Laboratories, Richmond, CA), as described before [42].

### Construction of clean-deletion mutants and overexpression plasmids

Non-polar and in-frame deletion mutations were introduced into *L. lactis* NZ900 using, essentially, the method described by Solem *et al.* [43]. The pCS1966-derived vectors listed in Table 5 were constructed by consecutively cloning the two flanking regions of a given gene, obtained by PCR with the specific primer pairs P1/P2 and P3/P4, presented in Table S1. Since pCS1966 does not replicate in *L. lactis*, all intermediate cloning steps were performed in *E. coli* DH5α (grown in TY broth, with 120 µg ml^−1^ Em when required). Overexpression of *ftsH*, *oxaA2*, *cesSR*, *rmaB* and *llmg_2163* was achieved via read-through from the *repA* promoter in pIL252 and pIL253 [27]. PCR fragments containing these genes and their native ribosome binding site, were obtained using the Forw/Rev primer pairs indicated in Table S1 and cloned into pIL252 and pIL253, yielding the respective derivatives depicted in Table 5.

### DNA-microarrays

RNA was isolated from two biological replicates of both the experimental and the control cultures. An equivalent of 10 OD_600_ units (OD_600_[-] times sample volume [mL]) of *L. lactis* cells was harvested by centrifugation (1 min; 10,000 rcf; 4 °C). Pellets were immediately frozen in liquid nitrogen and kept at −80 °C until further processing. Cells were resuspended in 500 µl TE (10 mM Tris-HCl; pH 8.0; 1 mM EDTA) and transferred to 2 mL screw-capped tubes, after which 50 µl 10% SDS, 500 µl phenol/chloroform, 500 mg glass beads (50–105 µm of diameter) and 175 µl macaloid suspension (Bentone, Hightstown, NJ) were added. The macaloid suspension was prepared as follows: 2 g macaloid were added to 100 ml TE, boiled during 5 min, cooled to room temperature, sonicated during short periods of time until a gel was formed, spun down and resuspended in 50 ml TE (pH 8.0). All reagents used for RNA work were treated with DEPC (diethyl pyrocarbonate; Sigma-Aldrich). Cells were disrupted by bead-beating twice during 45 s (with a cooling step on ice during 1 min in between) in a Mini-Bead Beater (Biospec Products, Bartlesville, OK). The cell lysate was cleared by centrifugation (10 min; 11,000 rcf; 4 °C) and 500 µl supernatant was extracted with 500 µl phenol/chloroform, and subsequently with 500 µl chloroform. Total RNA was isolated from the water phase using the High Pure RNA Isolation Kit (Roche Molecular Biochemicals, Mannheim, Germany) according to the manufacturer's protocol. RNA quality was verified in an Agilent Bioanalyzer 2100 using RNA 6000 LabChips (Agilent Technologies Netherlands BV, Amstelveen, the Netherlands) and RNA concentration was determined spectrophotometrically in a Nanodrop ND1000 (NanoDrop Technologies, Wilmington, DE). Copy DNA (cDNA) was synthesized using Superscript III Reverse Transcriptase (Invitrogen, Carlsbad, CA) and amino allyl-modified dUTP's in the nucleotide mix. Indirect Cy-3/Cy-5 labelling of cDNA was performed according to supplier's instructions (Amersham Biosciences, Piscataway, NJ). Hybridisation of Cy-labelled cDNA during 16 h at 45°C in a microarray hybridisation incubator ISO20 (Grant Boekel, Cambridgeshire, UK) was performed with Ambion Slidehyb #1 hybridisation buffer (Ambion Biosystems, Foster City, CA) on mixed amplicon and oligonucleotide spotted SuperAmine (ArrayIt, Sunnyvale, CA) glass slides. These array slides cover 2308 of the 2435 predicted ORFs from *L. lactis* subsp. *cremoris* MG1363. Slides were scanned using a GenePix Autoloader 4200AL scanner (Molecular Devices Corporation, Sunnyvale, CA). DNA microarray data from biological replicates were obtained with dye-swaps, to discard possible differences between the Cy-3 and Cy-5 labelling reactions. Slide images were analysed using ArrayPro 4.5 (Media Cybernetics, Silver Spring, MD). Slide data were processed and normalized using MicroPrep [44], [45]. Differential expression tests were performed with the Cyber-T implementation of a variant of Student's *t*-test [46]. Only operons in which at least one gene whose expression ratio was associated with a Bayesian *p*-value lower than 0.0001 were considered for further analysis.

All data is MIAME compliant and has been deposited in GEO [47].

### Protein Techniques and Flow Cytometry

Membrane vesicles were prepared essentially as described by Poolman et al., [48], with the following modifications: 10 OD_660_ units of an *L. lactis* culture was pelleted and resuspended in 1 mL of 50 mM potassium phosphate, pH 7.0; the suspension of cells was bead-beated twice (as described above) after which it was cleared by centrifugation (10 min; 11,000 rcf; 4°C). The supernatant was subsequently centrifuged (45 min; 80,000 rpm; 4°C) using a TLA-120.1 rotor in an Optima TLX Ultracentrifuge (Beckman, Fullerton, CA). The pellet was resuspended in loading buffer and used for SDS-(10%)PAGE analysis. Wet-blotting (16 h; 24 V; 4°C) of SDS-PAGE separated proteins was performed with a Mini Trans-Blot (Bio-Rad Laboratories). Immunodetection was performed using Anti-His conjugated antibodies (Qiagen, Hilden, Germany) according to manufacturers' instructions. Visual quantification of protein bands on coomassie-stained SDS-PAA gels was performed with the Quantity One software (Bio-Rad Laboratories). Quantification of BcaP-GFP-H6 production was determined by whole-cell-fluorescence using an Epics XL-MCL flow-cytometer (Coulter, Fullerton, CA) using the WinMDI 2.8 software (http://facs.scripps.edu/software.html). β-galactosidase activity assays were performed on cell suspensions as described previously [49].

## Supporting Information

Figure S1 **Functional production of BcaP and its derivatives in** ***L. lactis*** **MG1363Δ** ***bcaP*** **.** Strains were grown at 30°C in SA medium with 5 µg ml-1 chloramphenicol, when required, and all 20 amino acids. BcaP (full squares), BcaP-H6 (full diamonds) or BcaP-GFP-H6 (full triangles) were induced in either strain with 5 ng/m of nisin A at an OD600 = 0.4. The uncomplemented *L. lactis* MG1363Δ*bcaP* is represented by a dotted line with empty circles and the uncomplemented wild-type *L. lactis* MG1363 by a dotted line with full circles.
(TIF)

Table S1 **Oligonucleotides used in this study.**
(DOC)
