# Supplementary figures and images for: Efficient Overproduction of Membrane Proteins in Lactococcus lactis Requires the Cell Envelope Stress Sensor/Regulator Couple CesSR
Source: PLoS One. 2011 Jul 19;6(7):e21873. doi: 10.1371/journal.pone.0021873 (PMC3139573; doi:10.1371/journal.pone.0021873)

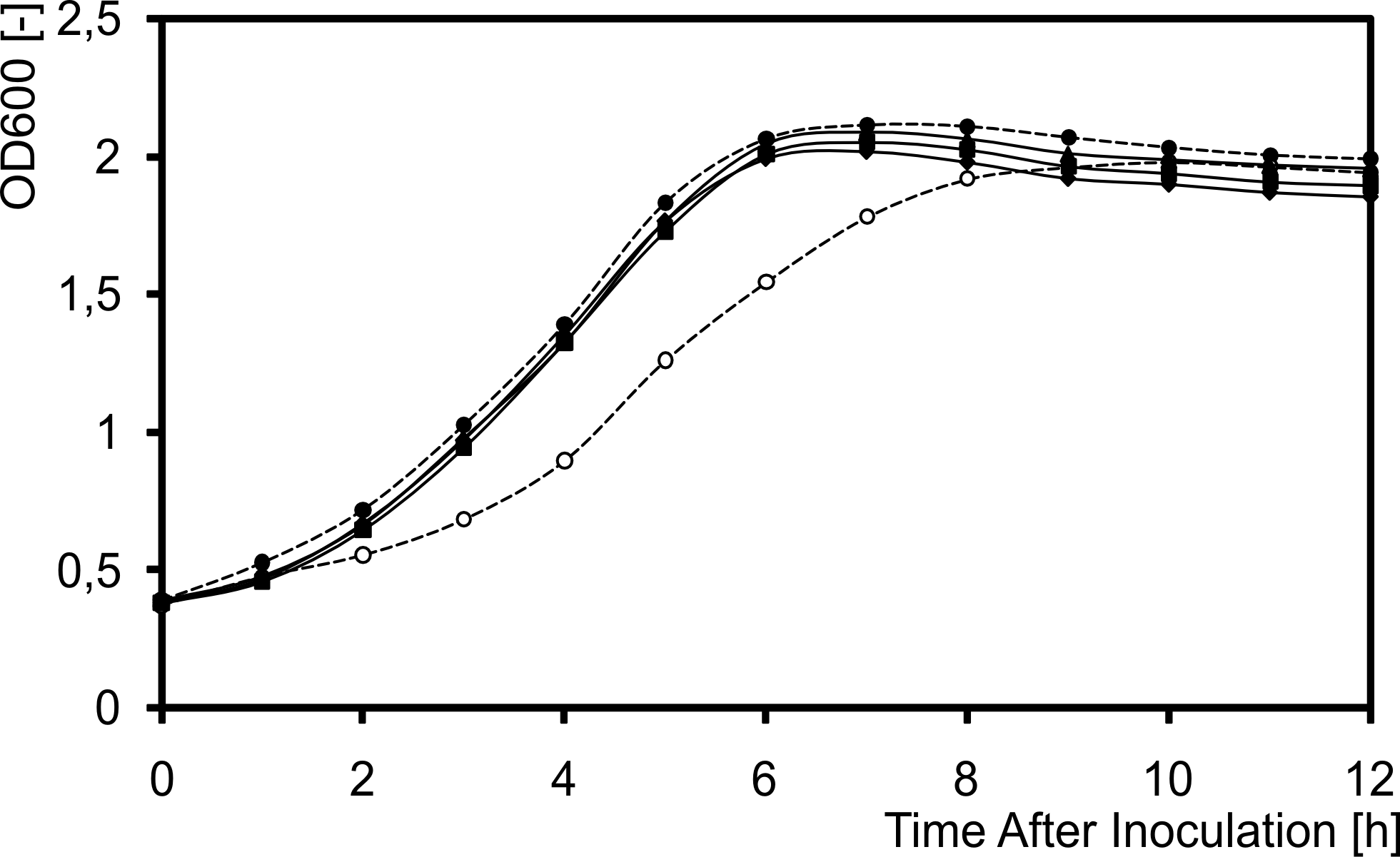

Supplement: Figure S1 — Functional production of BcaP and its derivatives in L. lactis MG1363Δ bcaP . Strains were grown at 30°C in SA medium with 5 µg ml-1 chloramphenicol, when required, and all 20 amino acids. BcaP (full squares), BcaP-H6 (full diamonds) or BcaP-GFP-H6 (full triangles) were induced in either strain with 5 ng/m of nisin A at an OD600 = 0.4. The uncomplemented L. lactis MG1363ΔbcaP is represented by a dotted line with empty circles and the uncomplemented wild-type L. lactis MG1363 by a dotted line with full circles. (TIF) [file pone.0021873.s001.tif]
