# Supplementary material for: Efficient Overproduction of Membrane Proteins in Lactococcus lactis Requires the Cell Envelope Stress Sensor/Regulator Couple CesSR
Source: PLoS One. 2011 Jul 19;6(7):e21873. doi: 10.1371/journal.pone.0021873 (PMC3139573; doi:10.1371/journal.pone.0021873)
Supplement: Table S1 — Oligonucleotides used in this study. (DOC) [file pone.0021873.s002.doc]

**Table S1: Oligonucleotides used in this study.**

| Name | Nucleotide Sequence (5’ > 3’; restriction enzyme sites and hexahistidine tags are underlined) |
| --- | --- |
| P1-*llmg_0021* | GGAATTCTAGATGGGTCAGTTCCTTTCTTAGC |
| P2-*llmg_0021* | CATTAGGATCCTTTTTACAAAATTTCCTTGTCTTGG |
| P3-*llmg_0021* | AATGCGGATCCAAGCAGCTGCAGCAGAAGTACC |
| P4-*llmg_0021* | CTTCCCTCGAGATAGGACTTGACTTCTGTGTTCG |
| P1-*llmg_0165* | AAGGTTCTAGAAAAGGTGGGCATTCATCAAAGG |
| P2-*llmg_0165* | CCGAAGGATCCACGTAAGGCTCCCTTGGCTTG |
| P3-*llmg_0165* | GGACAGGATCCTACCTCAATATGGCTTCAATTGC |
| P4-*llmg_0165* | TCTTGCTCGAGCTCCGTCTAGTACACGGTGTTGG |
| P1-*llmg_0169* | AAAATTCTAGAGACAGCTGGCTTGCTAATCATCC |
| P2-*llmg_0169* | AAGCGGGATCCGTACGATTATTCCTGACAAAATTCC |
| P3-*llmg_0169* | AGTCCGGATCCTACTTGGTAAAATGAAAAATTAAGCAGC |
| P4-*llmg_0169* | GTGCTCTCGAGGAGTAGTAGGCCACCGAGTAACC |
| P1-*llmg_0540* | AAAAGGGATCCAATCTGGTGGACGAAATGCTGG |
| P2-*llmg_0540* | GAGTAGGATCCCGGCTGTCATTAAACCAGTCAATG |
| P3-*llmg_0540* | CTCAGTCTAGAGTCAAAAAGCTGGGGTTGAAGC |
| P4-*llmg_0540* | ATAAACTCGAGGCTTGGGGCTGTGCCAAAGC |
| P1-*llmg_1103* | CCCGAGCGGCCGCAAAAGGCGGCCAAACAAAAGC |
| P2-llmg_1103 | CATTGGGATCCCAAAGCCCCACCGAGTGAACC |
| P3-*llmg_1103* | GTCCTGGATCCGGCAAACCCTTTGTCGCTAAAGC |
| P4-*llmg_1103* | GCAGTCTCGAGAAAAGGTTGAACAGCACCAACC |
| P1-*llmg_1115/6* | TGTCGTCTAGAGGTCATGTGTCCCTTGTTCAAGC |
| P2-*llmg_1115/6* | AAAATGGATCCCACGATTCCTAGATGACCATAACC |
| P3-*llmg_1115/6* | TATCCGGATCCCCATGCGTATTCAAGCCGAAGG |
| P4-*llmg_1115/6* | TTAAACTCGAGCCAACCATCCCTGGTTCTTGG |
| P1-*llmg_1649/8* | TTTCTTCTAGAATTACTGACAGATATTTCATCAC |
| P2-*llmg_1649/8* | ACTGCGGATCCGGTTGTGAAAAGCGATTTTTTCATCC |
| P3-*llmg_1649/8* | TCAATGGATCCTAAGTTTCGGACCGCACTCAGGC |
| P4-*llmg_1649/8* | ATTGGCTCGAGTAATTGCTGTGGTGCCTTTGTAG |
| P1-*llmg_1860* | GGAGATCTAGATTCTGGAGGAGAGCGACAACG |
| P2-*llmg_1860* | CTGCTGGATCCGCAAATAGAACGCTTGGATTGTGC |
| P3-*llmg_1860* | CGTCCGGATCCTGATGAATTTGACCCTAGAGC |
| P4-*llmg_1860* | TCTGACTCGAGTTGAAATTTTTCATTACTTCTCG |
| P1-*llmg_1918* | AATTTGCGGCCGCATTGCTCAAAATGTGAGCGGTTC |
| P2-*llmg_1918* | ACTAAGGATCCCATTCAATCCGTCGCTTCGTTGG |
| P3-*llmg_1918* | TGATTGGATCCACATGTTCCTCTTCCTGCTCC |
| P4-*llmg_1918* | GGCATCTCGAGCAATATGGCGCATACGGGTCTG |
| P1-*llmg_2163* | TGGTGTCTAGACACTCGTTAACGGAAATATCTTGC |
| P2-*llmg_2163* | CAATGGGATCCCTCACTCGACGATTTGTCACC |
| P3-*llmg_2163* | CCAGTGGATCCAGAAAAAGAAGATGACTGGTCAG |
| P4-*llmg_2163* | CTAATCTCGAGATACTCCGTAGACAATTGAAAGTC |
| Forw-*llmg_0021* | TTTTCGGATCCTAAATAAACAAAAGATAAGGAAAATATGAATAAC |
| Rev-*llmg_0021* | TCTAATCTAGATTAGTGATGGTGATGGTGATGATTGGCTGTTTCATCTTCTGATTTTTCC |
| Forw-*llmg_0540* | GGTAAGGATCCTTAATTTATAGAAGGTGAACTTATATTG |
| Rev-*llmg_0540* | AGTAATCTAGATTAGTGATGGTGATGGTGATGCTTACGATTTTGTTTACCAGCATTTCG |
| Forw-*llmg_1649/8* | TCGTGGGATCCCAGGAAATATTGAGGTGATTAGAGGATG |
| Rev-*llmg_1649/8* | TCATATCTAGATTAGTGATGGTGATGGTGATGTACTAAATGATGTTGAATGGCATAAATAG |
| Forw-*llmg_1860* | CAACCGGATCCAACTAAGGAGAACAAATTATGTCAGAAC |
| Rev-*llmg_1860* | CATCTTCTAGATTAGTGATGGTGATGGTGATGGAAATCATCCCAGTTTTCTGGTTTTTTAGG |
| Forw-*llmg_2163* | GATAAGGATCCTATTAAAAGAAAGGATAAATTATGTCTC |
| Rev-*llmg_2163* | TGAACTCTAGATTAGTGATGGTGATGGTGATGAAAATCTGACCAGTCATCTTCTTTTTC |
